# Supplementary material for: Flexible Supercapacitors Based on Graphene/Boron Nitride Nanosheets Electrodes and PVA/PEIGel Electrolytes
Source: Materials (Basel). 2021 Apr 14;14(8):1955. doi: 10.3390/ma14081955 (PMC8069789; doi:10.3390/ma14081955)
Supplement: Supplementary file 1 [file materials-14-01955-s001.pdf]

materials

Supplementary Materials

Flexible Supercapacitors Based on Graphene/Boron Nitride Nanosheets Electrodes and PVA/PEI Gel Electrolytes

Chan Wang [1,2], Kuan Hu [3], Ying Liu [1,2], Ming-Rong Zhang [3], Zhiwei Wang [1,2] and Zhou Li [1,2,*]

[1] CAS Center for Excellence in Nanoscience, Beijing Key Laboratory of Micro-Nano Energy and Sensor, Beijing Institute of Nanoenergy and Nanosystems, Chinese Academy of Sciences, Beijing 101400, China; wangchan@binn.cas.cn (C.W.); liuying@binn.cas.cn (Y.L.); wangzhiwei@binn.cas.cn (Z.W.)
[2] School of Nanoscience and Technology, University of Chinese Academy of Sciences, Beijing 101400, China
[3] Department of Advanced Nuclear Medicine Sciences, The National Institute of Radiological Sciences, The National Institutes for Quantum and Radiological Science and Technology, Chiba 263-8555, Japan; kuan.hu@qst.go.jp (K.H.); zhang.ming-rong@qst.go.jp (M.-R.Z.)
* Correspondence: zli@binn.cas.cn

Citation: Wang, C.; Hu, K.; Liu, Y.; Zhang, M.-R.; Wang, Z.; Li, Z. Flexible Supercapacitors Based on Graphene/Boron Nitride Nanosheets Electrodes and PVA/PEI Gel Electrolytes. *Materials* **2021**, *14*, 1955. https://doi.org/10.3390/ma14081955

Academic Editor: Antonino Salvatore Aricò

Received: 12 March 2021
Accepted: 12 April 2021
Published: 14 April 2021

Publisher's Note: MDPI stays neutral with regard to jurisdictional claims in published maps and institutional affiliations.

Copyright: © 2021 by the authors. Licensee MDPI, Basel, Switzerland. This article is an open access article distributed under the terms and conditions of the Creative Commons Attribution (CC BY) license (http://creativecommons.org/licenses/by/4.0/).

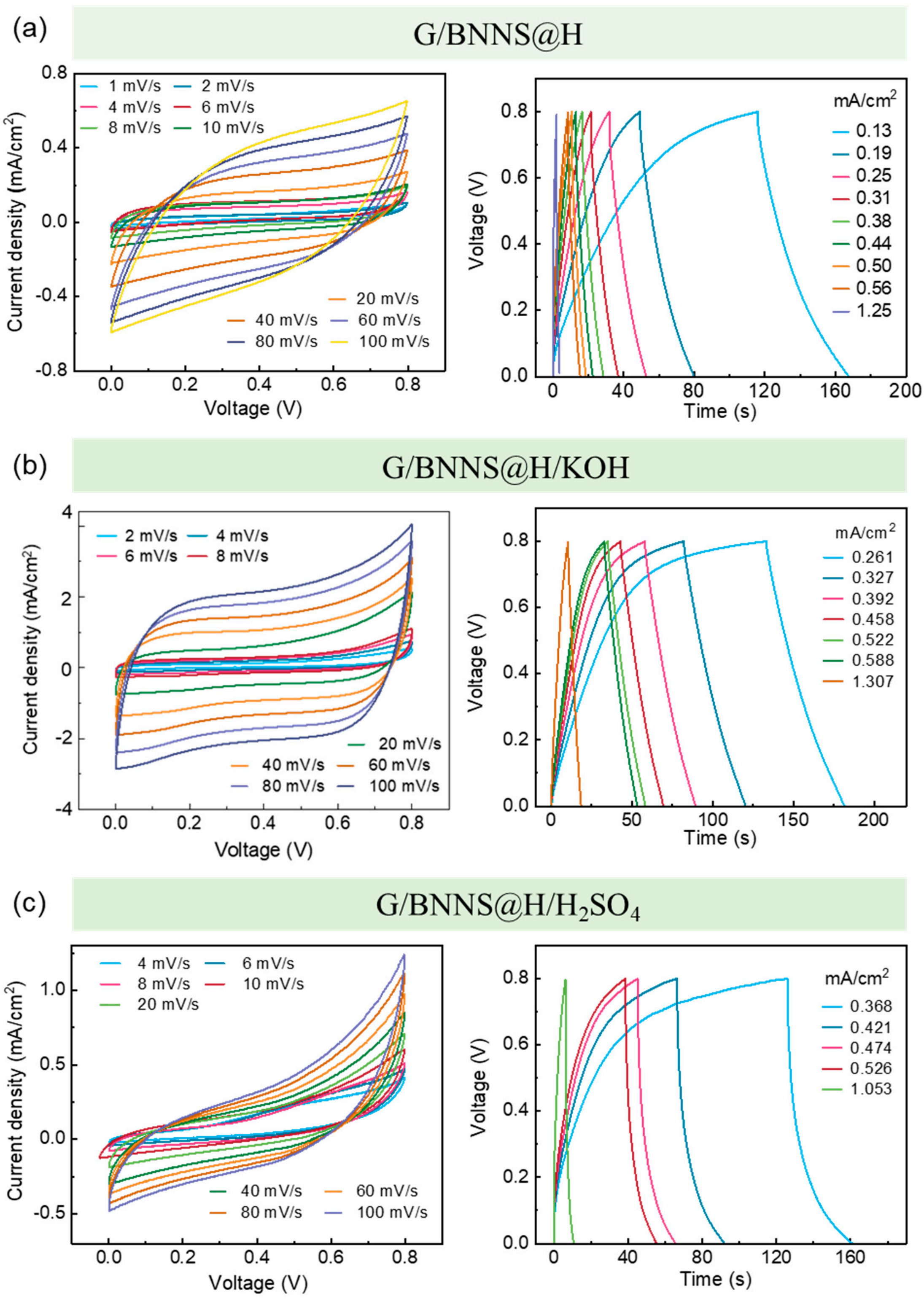

Figure S1. (**a**) The CV and GCD curves of the supercapacitor based on G/BNNS@H. (**b**) The CV and GCD curves of the supercapacitor based on G/BNNS@H/KOH. (**c**) The CV and GCD curves of the supercapacitor based on G/BNNS@H/H_2SO_4.

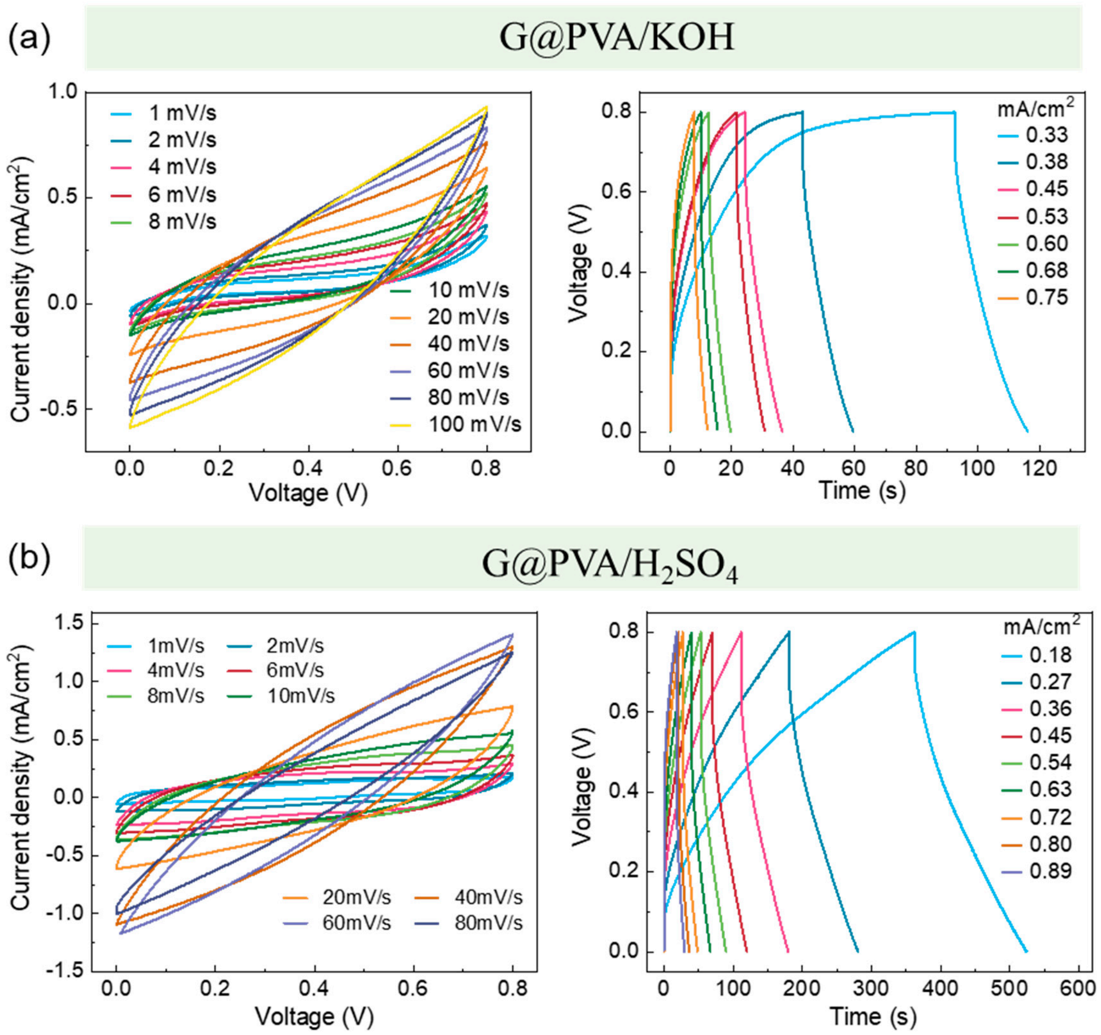

Figure S2. (**a**) The CV and GCD curves of the supercapacitor based on G@PVA/KOH. (**b**) The CV and GCD curves of the supercapacitor based on G@PVA/H_2SO_4.

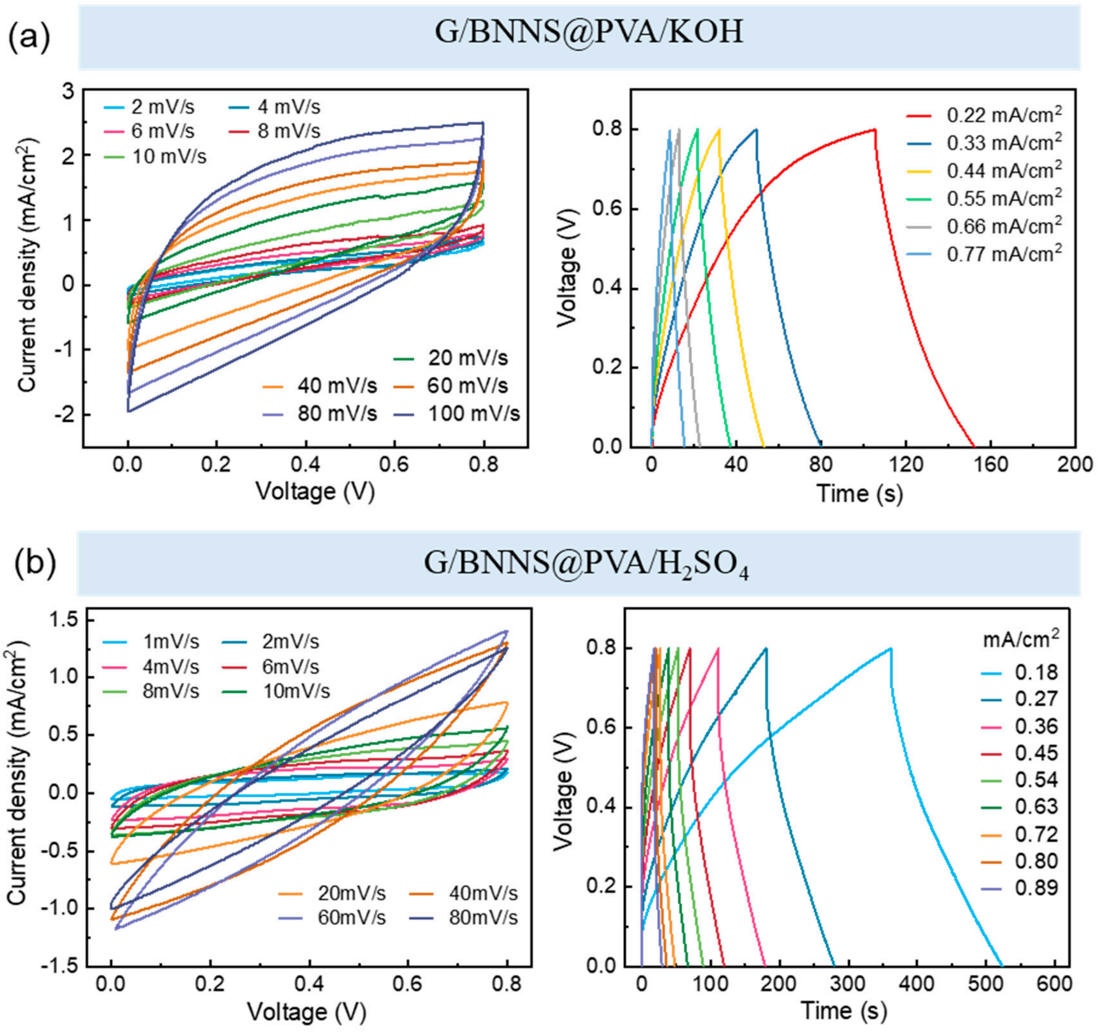

Figure S3. (**a**) The CV and GCD curves of the supercapacitor based on G/BNNS@PVA/KOH. (**b**) The CV and GCD curves of the supercapacitor based on G/BNNS@PVA/H_2SO_4.
